# Hospital Expenditure—The Commissariat

**Published:** 1896-12-19

**Authors:** 


### THE INSTITUTIONAL WORKSHOP.

### HOSPITAL EXPENDITURE—THE COMMISSARIAT.

### XXXIII.—THE PREVENTION OF WASTE.

The more than common impediments to economy existing in hospitals are due in great measure to the varied composition of the household. Each department is necessarily to some extent under distinct management, and those who actually arrange the bill of fare and administer the supplies are often entirely unconcerned with the cost. No true economy can ever be expected under a system by which the persons who order and use supplies and the person who is responsible for the bills both work independently and pull different ways. Such a system is to be seen often enough in private life in houses where all orders to the tradesmen are given by the cook, and where the mistress reserves to herself merely the duty of looking through the bills and obtaining the necessary money to pay them from the master of the supplies. In many institutions the conditions of management at this moment are not dissimilar to this. For cook substitute matron; the secretary takes the place of nominal responsibility occupied by the mistress of the household, and in the background looms the stern paterfamilias in the person of the general committee. Nothing can be more unsatisfactory.

It is absolutely essential to a well-balanced administration that a spirit of responsibility should permeate the whole establishment, from the manager on whom the multifarious charges accumulate for payment, down to the youngest probationer charged with serving out the rice pudding in her ward. And this spirit of responsibility must in all cases recognise a double duty. There is the duty towards those for whom each person, invested with authority of any kind, is called on to provide, and there is the duty towards the institution represented by the person next in authority. The doctor, for example, in ordering "extras," is responsible for the patients' welfare, but he is also responsible to the institution that no undue favour shall be shown to one more than another, or to one man's patients more than to those of another. Again, it is the sister's duty to see that the patients in her ward have their fair allowance of milk, beef-tea, &c., sent up in good condition; it is also her duty to the institution to take extreme care that no more is sent than is needed, and that none is improperly used. The matron has a duty to the nurses to see that their table is amply and comfortably provided; but she owes it also to the institution to cater for them economically, and to avoid all occasions of waste. Where either of these equally-important duties of responsibility is allowed to slumber, the institution suffers. If the sense of responsibility is dimmed towards those in subordination, a sense of officialism reigns supreme, and the comfort of every inmate is sacrificed to a rampant code of unwritten regulations. If, on the other hand, no sense of responsibility is cultivated towards the institution as a whole, the management degenerates into a silent but incessant struggle among those in authority over the different departments to secure the largest share for their own particular charges. Where this dual sense of responsibility is nicely balanced in every member of the institution, the management may be considered perfect.

To that end it is above all things necessary that there should be one person invested with general control, and endued with a grasp of the whole situation. In small hospitals this may be the matron, in large ones it may be the secretary, steward, or manager; but whatever the name or sex, the duties of the person filling this important post, however they may vary in detail, are in effect the same, viz., to understand and control each separate strand of expenditure, and so balance and direct the whole that none may predominate, while none is allowed to come short.

The perfect manager, like many a clever housewife, will have no direct control of the purse. Like such an one he may be lacking in power to draw a single cheque without reason shown, and must submit the result of his work in the minutest particulars of expenditure to those who grant the supplies. Yet, none the less, his is the eye which supervises the whole, and his is the hand to which all turn in the constantly-recurring need in institution life of "getting things done." He knows, for example, on which floor the waste in electric light took place the week before last, and can bring the charge home to the possibly unconscious offender with array of indisputable figures. He knows that the new sister is too free-handed in the matter of milk, and that a certain surgeon is growing laxer in the use of extras. He knows about the tough meat, and has taken steps to prevent its recurrence, long before the complaints reach him from the doctor's table, and it is he who detects that the butterman is supplying short weight. He notes that the sash-line of the scullery window is broken, and the hinge of the office coal-box is not beneath his attention. He never interferes in the usual sense of the word, for interference is but a symptom of partial knowledge. It is, in brief, his business to know just a little more about each department than those actually in charge.

It may be asked how, short of a hateful system of espionage, is this knowledge to be attained in establishments the size of a small town, containing such complex elements as exist in the hospital; and even when the knowledge is attained, how can it be turned to practical account among so many persons of independent status, admitting of no divided sway in their several spheres?

The most satisfactory way to solve doubts such as these would be to pay a visit to one of those hospitals such as Guy's, the Middlesex, the Birmingham General Infirmary, and many others which could be named, where the desired results are obtained by systems differing perhaps in every detail but identical in principle. The principle on which all hinges is briefly that all who use supplies of any kind shall furnish signed orders, daily, weekly, or monthly, according to the nature of the article, stating what is required in each department and for how many persons; and that the central authority, having collected and checked such orders, shall combine them into returns revealing without possibility of mistake the slightest variation in the normal rate of consumption of each article, and the exact department to which such variations may be attributed. Some of the returns thus prepared, such as that giving an account of lint, surgical necessaries, used by each doctor, or of crockery, brooms, &c., supplied to each ward, are circulated back again among those responsible for the use of these articles in order that each may compare his or her rate of expenditure with that of others similarly situated. It cannot be too often repeated that in these matters, to know is to control. There is no other road to economy. It cannot be compassed by appeals to conscience, not by nagging, not (certainly not) by a grudging, stingy giving out of necessary articles, as though the consumer were guilty in requiring them. Let each housemaid know how many yards of house flannel she is expected to use in a given time; let each pound of lint be registered against someone's name who shall be accountable; let each article be given out at the right time, on the right day, to the right person, and with the understanding that the transaction, slight though it may be, has been noted down, and will duly find its place in the weekly average and in the record of the whole expenditure. Let such a system once take root, and then, be the institution large or small, rich or poor, hampered with old traditions, or brimming over with untried theories, a spirit of true economy will reign through every department, worth far more, indeed, than the trifling secretarial labour expended in its production.

It may be objected that in small institutions containing less than a hundred persons, such a system, if not impracticable, would carry far less weight than where the range of comparison is wider. But though this may be true, it must be remembered that in small hospitals it is possible to initiate those who administer the supplies into the mysteries of the housekeeping in a manner which could not be thought of in large institutions. One of the cleverest provincial matrons of the day, who combines in her own person the complex duties of manager, housekeeper, and matron, is in the habit of allowing each sister in turn to act for a week occasionally as housekeeper under her direct supervision, and it cannot be doubted that such an experience of the expense and difficulties of the catering would have a valuable effect in promoting a sense of responsibility in the use of ordinary articles.

We propose to give one or two specimens of the way in which returns, such as we have indicated, may be prepared in small, as well as large households.

